# Retention Force Comparison between Hawley and Vacuum-Formed Retainers in Orthodontic Treatment of Maxillary Arch: A Prospective, Non-randomized, Cohort Study over 6 Months: Comparing Retention Force of Hawley and Vacuum Formed Retainers

**DOI:** 10.31661/gmj.v13iSP1.3592

**Published:** 2024-12-25

**Authors:** Erfan Asadolahi, Shabnam Saleh, Yashar Rezaei, Mortaza Hosenzadegan, Ahmad Behroozian, Reza Sharifi

**Affiliations:** ^1^ Department of Orthodontics, Tabriz University of Medical Sciences, Tabriz, Iran; ^2^ Department of Dentistry, Belarusian State Medical University, Minsk, Belarus; ^3^ Department of Dental Biomaterials, Faculty of Dentistry, Tabriz University of Medical Sciences, Tabriz, Iran; ^4^ Department of Prosthodontics, Faculty of Dentistry, Tabriz University of Medical Sciences, Tabriz, Iran

**Keywords:** Retention Force, Hawley Retainers, Vacuum-Formed Retainers

## Abstract

**Background:**

This study aimed to investigate the change in the retention force of Hawley and vacuum-formed retainers for the maxillary arch over a 6-month period.

**Materials and Methods:**

In this prospective, non-randomized cohort study, 50 patients who were prescribed Hawley or Vacuum-formed retainers for their maxillary arch were consecutively enrolled. Retainers were fabricated to the standardized design on casts and were exclusively checked for every patient. The retention force of retainers was evaluated based on the force (in Grams) required to remove them from the mouth. Retention force was assessed on delivery day, 3 and 6 months after treatment via force gauge. The changes in retention force from one timepoint to another were calculated as a ratio (retention change index) and were then compared between two retainers.

**Results:**

Forty-five patients (23 Hawley and 22 vacuum-formed) completed the study. The retention force of Hawley retainers throughout three-time points was 453-249-189 (g), while that of vacuum-formed retainers was 857-621-513 (g). The decrease in retention force was statistically significant for both retainers (P-value 0.001). The retention force decrease in Hawley retainers was significantly more than Vacuum-formed retainers throughout 6 months (P-value 0.05).

**Conclusion:**

Both retainers experienced a loss in retention force over the 6-month period. However, vacuum-formed retainers maintained a higher retention force compared to Hawley retainers, making them more effective in retaining the teeth in their corrected positions.

## Introduction

One of the greatest concerns after the orthodontic treatment is the relapse of the teeth to their original position. According to previous studies, up to 70 percent of patients experience it after the treatment, and it is more likely to happen following the first two years post-treatment [1]. A retention force is required after the treatment to maintain the teeth at their new position. Retention is provided by orthodontic retainers, which can be removable or fixed. Fixed retainers, also known as bonded retainers, are usually bonded to the teeth via an integrated wire [2]. They do not need patient cooperation; they are more effective than removable retainers and are suitable for lifelong retention. However, they come with some drawbacks such as precise bonding technique, fragility, and a tendency to cause periodontal problems by weakening oral hygiene [3]. Two common removable retainers are Hawley retainer (HR) and Vacuum-formed retainer (VFR). HR was designed by Charles Hawley in 1919 [4]; it is an adjustable plaque including claps on the molars and a labial bow from canine to canine, which has been used as an effective retainer for almost a century [5]. HR are typically made of acrylic and wire and are designed to maintain occlusal contacts and prevent tooth movement [6]. VFR, also known as clear overlay retainer or Essix was designed by Ponitz in 1971 [6]. It is removable, and transparent and has become more popular than HRs because they are more esthetic, have lower costs, and are easier to fabricate [7]. VFR advantages over HR, especially in terms of speech and comfort, are mainly due to the absence of palatal coverage [8]. Numerous studies have already compared the characteristics of Hawley and vacuum-formed retainers in terms of periodontal health and compliance [9], the force of biting [10], durability [11][12], cost-effectiveness [13], survival of retainers [14] and occlusal contacts [10][15]. Another important factor affecting the efficiency of orthodontic retainers is their ability to withstand forces encountered during daily activities, such as chewing, speaking, and facial movements. Insufficient retention force can lead to premature failure of the retainer, which leads to displacement or removal of the retainer from the teeth; this becomes more accentuated for upper arch removable retainers. This point ultimately breeds discomfort and inconvenience for patients [12][13][14][15]. Retention force in orthodontics refers to the forces that act on the teeth during the retention phase of orthodontic treatment, which is necessary to prevent relapse of the final occlusal outcome. These forces can come from the periodontal fibers around the teeth, which tend to pull the teeth back toward their pre-treatment positions, as well as from deflecting occlusal contacts if the final occlusion is less than ideal [16]. While a number of studies have evaluated the patient’s satisfaction [17] and conveniences [18] of the Hawley and Essix retainers, limited studies have clinically investigated the retention force of removable retainers over a period of time. A systematic review of randomized controlled trials found that VFRs were more effective than Hawley retainers in maintaining arch dimensions and alignment [19]. Another study found that VFRs were more effective than Hawley retainers in preventing tooth rotation and maintaining intercanine and intermolar widths [20]. However, a meta-analysis found that Hawley retainers had better periodontal health compared to VFRs [21]. As previous studies have primarily focused on the comparison of Hawley and vacuum-formed retainers in terms of periodontal health, compliance, and durability, with limited attention to their retention force over time, we aimed to investigate the change in retention force of these two types of retainers for the maxillary arch over a 6-month period. Moreover, the existing literature has shown inconsistent results regarding the effectiveness of Hawley and vacuum-formed retainers in maintaining arch dimensions and alignment, with some studies suggesting that vacuum-formed retainers are more effective, while others found Hawley retainers to have better periodontal health.

## Material and Methods

### Study design, population, and ethics

This was a prospective, non-randomized, clinical study conducted on 50 patients who were referred to the university clinic of dentistry school, Tabriz, Iran from October 2022 to May 2023. All patients had completed their orthodontic treatment of maxillary arch and were required to start using removable retainers (Hawley or Vacuum-formed). The procedures and protocols of this study in terms of the protection of human subjects were approved by the Research Ethics Committee of Tabriz University of Medical Sciences (IR.TBZMED.REC.1401.1028). All patients were fully informed about the goals of the study, and the ones who agreed with the conditions, all signed a written consent.

The inclusion criteria for the study were the completion of fixed orthodontic treatment, availability for at least 6 months, absence of any systemic disease, no gingival inflammation or infection, and not being a heavy smoker (defined as smoking 3-5 cigarettes per day). The exclusion criteria included pregnancy, allergic reaction to acrylic resin, any damage or trauma to the maxillary arch, and not being cooperative with the instructions.

A priori power analysis was conducted using G*Power software (version 3.1.9.2) to determine the required sample size for each group. Based on the expected difference in means of hardness between the groups based on the study of Aldweesh et al. [22], we calculated the required sample size to achieve a power of 0.8 and an alpha level of 0.05 to be approximately 25 persons per group.

### Treatment preparation and retainer prescription

**Figure-1:**
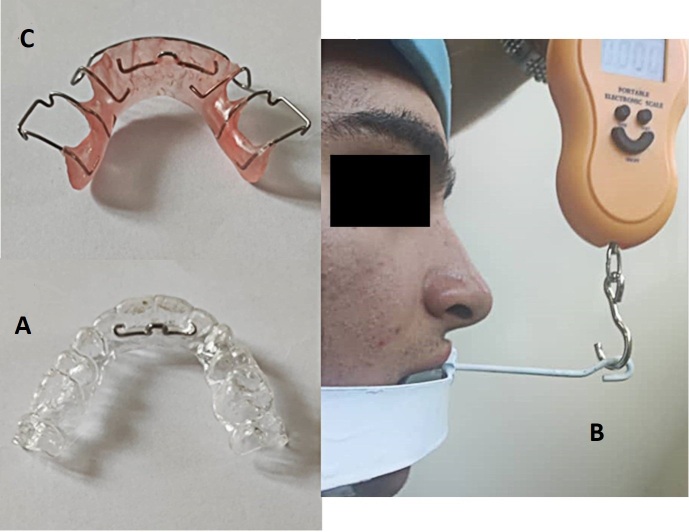
Figure 1. A) Hawley retainer. B) Vacuum-formed retainer. C) Force gauge and head holder.

**Figure-2:**
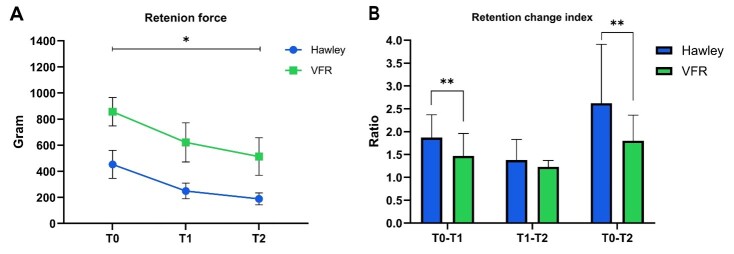
Figure 2.A) The amount of force (in grams) required to remove the retainers from the mouth in T0, T1 and T2. B) The ratio of change in retainers’ retention force from one timepoint to another (T0-T1, T1-T2 and T0-T2). Data are presented in Mean ± SD. VFR: Vacuum-formed retainer. T0: immediately after retainer delivery; T1: 3 months after delivery; T2: 6 months after delivery. *: Mean differences throughout T0, T1 and T2 are statistically significant (Repeated measure ANOVA; P-value < 0.001 for both Hawley and VFR). **: Mean differences between Hawley and VFR are statistically significant (Two-tailed T test; P-value = 0.015 for T0-T1; P-value = 0.013 for T0-T2).

No intervention was applied on retainer selection for patients; they were prescribed either Hawley or vacuum-formed retainers according to the standard practice protocol by their orthodontist [23]. Patients were then recruited consecutively until the sample size was attained (25 Hawley and 25 vacuum-formed). On the debonding day, the maxillary fixed retainers were removed, following which alginate impressions (Tropicalgin Zhermark; Italy) were taken for modeling maxillary arch casts. Next, vacuum-formed retainers (Figure-1A) were fabricated with 1 thermoplastic platen (3A Co.; Korea), while Hawley retainers (Figure-2A) were composed of Adams Clasp on the first molar teeth, labial bow with 28-mil SS wire (Dentaurum, Germany) and acrylic resin (Dentaurum; Germany). All retainers were fitted on the casts to make sure they were flawless and without any damage; additionally, the retainers were checked on patients to ensure they were comfortable. Patients were instructed to wear their retainers full time (except for eating and brushing) for the first 3 months, and night-time (12 hours) for the second 3 months. In addition, all patients were sent messages every week, reminding them about the protocols and conditions of using retainers. The whole process of brackets removal, cast modeling, retainer fabrication, and delivering retainers took 1 week for every patient. Patients were recalled in 2 intervals, 3 months (T1) and 6 months (T2) after delivering removable retainers, to assess the retention force of their retainers.

### Retention force Assessment of retainers

The retention force of retainers was assessed according to the amount of force required to remove the retainer from the mouth; in other words, the more force required to remove the retainer, the more retention force the retainer had. To assess this force, patients were asked to place their heads on the head holder to maintain their heads in a static and completely horizontal position. Next, the force was applied via a force gauge through a hooked lever on the head holder to pull out the retainer (Figure-1B). The minimum force by which the retainer was removed from the mouth was recorded in grams. To prevent damage to the lower anterior teeth, a mouth guard was placed for them. In all patients, the head holder was positioned in alignment with their occlusal plane and was connected to their retainers through the holes that were designed in the retainers. All patients were evaluated at three timepoints: On the retainer delivery day (T0), 3 months after delivery (T1), and 6 months after delivery (T2).

### Retention change index

The amount of force required to remove the retainer from the mouth for VFR and Hawley retainers was different because of their different structure and positioning in the mouth and therefore it was not possible to compare them in terms of force. Instead, the amount of force change from one timepoint to another was recorded as a ratio and was then compared between Hawley and VFR retainers to indicate which one witnessed more change over the 6 months. Thus, the change ratio represents how much the retention force of each retainer reduces during 6 months. The retention change index is calculated as follows:


T1 - T2 = \frac{\text{Force}_{\text{(Grams)}} \text{ in T1}}{\text{Force}_{\text{(Grams)}} \text{ in T2}}

T0 - T2 = \frac{\text{Force}_{\text{(Grams)}} \text{ in T0}}{\text{Force}_{\text{(Grams)}} \text{ in T2}}

T0 - T1 = \frac{\text{Force}_{\text{(Grams)}} \text{ in T0}}{\text{Force}_{\text{(Grams)}} \text{ in T1}}

### Statistical analysis

Data analysis was carried out using Statical Package for Social Sciences (SPSS V.19). Quantitative variables were described as mean ± SD, while qualitative variables were presented as frequency (n) and percentage (%). Means between the two groups were compared with the Independent T-test (Parametric data) or Mann-Whitney U test (Non-parametric data). Repeated measure ANOVA was used to compare the means of each group throughout three-time points (T0, T1, and T2). P-value less than 0.05 was considered statistically significant. GraphPad Prism V.10 was used to illustrate charts.

## Results

**Table:** Table1. Demographic variables of patients receiving Hawley or VFR retainers

	**Hawley**	**VFR**	**P-value**
**Age, year**	21.83 ± 3.59	20.63 ± 2.87	0.227 ^*^
**Sex**			
**Male**	10 (62.5)	6 (37.5)	0.353 ^**^
**Female**	13 (44.8)	16 (55.2)	

Data are presented as Mean ± SD or frequency (%). **VFR** VFR: vacuum-form retainers.^*^ : Two-tailed T test; ^**^: Fisher exact test

### Patients and demographic variables

Fifty cases were invited to participate in the study. We lost contact with 2 of the patients, and 3 patients lost their retainers and therefore were excluded from the study. Overall, 45 patients (23 Hawley and 22 VFR) with a mean age of 21.24 ± 3.28 (Range: 16 - 30) were enrolled in the study. Demographic variables (age and sex) were not statistically different between Hawley and VFR groups and are presented in Table-1.

### Retainer retention force

The retention force of retainers was assessed according to the amount of force required to pull them out from the mouth at 3 different time points (Figure-1C), and the number of changes following each time point was recorded as the retention change index (Figure-1A). Overall, VFRs required more force to be removed from the mouth compared to the Hawley retainer, but both retainers experienced a steady reduction in their retention force over the 6 months. The reduction of force from T0 to T2 was statistically significant in both Hawley and VFR retainers (P-value < 0.001).

The retention force of Hawley retainers reduced 1.87 times, while the VFR retention force decreased 1.47 times from T0 to T1, and this difference was statistically significant (P-value = 0.015). The retention force of both retainers further reduced from T1 to T2, but, the changes (1.38 for Hawley and 1.23 for VFR) were less than those in T0-T1, and the difference between the retainers was not statistically significant (P-value = 0.16). Finally, retention force change over the 6 months (T0-T2) showed a greater reduction in Hawley retainers (2.62) in comparison to VFR (1.8), which was statistically significant (P-value = 0.013).

## Discussion

The retention phase is an important part of orthodontic treatment, which is provided by retainers, and has a crucial role in the prevention of relapse following treatment [24]. Previous studies have compared Hawley and VFR from different aspects. Two studies reported similar effectiveness in maintaining transverse expansion using HR and VFR [25][26]. Alkan et al. assessed the occlusal force distribution, individual tooth force, and occlusal surface area of HR and VFR and claimed that VRF was more effective than HR [27]. Ramezanzadeh et al. stated that VFR provided better retention than HR in two different protocols [11]; Demir et al. reported the same result in an in-vitro setting [12]. Moslemzadeh et al. reported that HR and VRF had a similar impact on periodontium; however, VRF is preferred as it is more esthetic [14]. Two systematic reviews in 2014 [19] and 2016 [28] have reported that there is insufficient evidence to show which retainer is more effective in terms of periodontal health, speech articulation, and orthodontic retention. One recent systematic review in 2020 [29] has also reported there is no evidence to show that the pattern of time duration wearing these retainers provides excellent stability. This study measured and compared the retention force of Hawley and vacuum-formed removable retainers over 6 months in clinical conditions.

Results showed that both retainers lost their retention force over 6 months up to 2.6 times (Hawley: 2.6 and VFR: 1.8) and the retention loss was higher in the first 3 months compared to the second 3 months for both retainers. Hawley, however, significantly lost more of its retention force compared to the VFR in the 6 months, and therefore VFR proved to be a better retainer in terms of retention force. The reason for its better retention force is that VFR is adapted to the mold by negative pressure, so the retainer is more precise and obtains its retention from teeth undercuts. Additionally, VFR is made of thermoplastic materials with elasticity properties, which makes it resistant to deformation. Hawley, on the other hand, is manually manufactured and therefore the retainer is less accurate and obtains its retention from Adam claps and labial bow [30]. Moreover, Hawley is made of acrylic and metal; acrylic shrinks in a moist environment and metal changes shape against the occlusal force [31].

The retention force of orthodontic removable retainers is of vital importance in ensuring an effective and comfortable orthodontic treatment. The results indicated retention loss for both retainers, which was more significant in the first 3 months. This suggests that the first 3 months after the orthodontic treatment with a removable retainer is important and requires more maintenance and follow-up for patients. Comfort and patient satisfaction with using retainers is important and therefore the retention strength of these retainers contributes to the long-term use and successful orthodontic treatment outcomes [21]. Limitations of our study are its short-term follow-up and single-center sample selection. Firstly, the non-randomized design of the study makes it challenging to establish cause-and-effect relationships between the type of retainer and the retention force, which may limit the generalizability of the findings. Additionally, the study’s design did not control for potential confounding variables, such as patient compliance, oral hygiene, or retainer wear and tear, which may have influenced the results. We believe there is a need to evaluate Hawley and VFR retainers over a longer period to indicate how their retention force change.

## Conclusion

In conclusion, both Hawley and VFR retainers witnessed a decline in their retention force over a 6-month period and the decline was significantly higher in the first 3 months. Furthermore, Hawley lost more retention force compared to VFR, which shows VFR has a better retention force compared to Hawley retainers.

## Conflict of Interest

The authors declare no conflict of interest.
